# Clinical Data Analysis for Treatment of Adult Inguinal Hernia by TAPP or TEP

**DOI:** 10.3389/fsurg.2022.900843

**Published:** 2022-05-20

**Authors:** Chunhui Cao, Xiaoyu Shi, Wei Jin, Fengming Luan

**Affiliations:** Department of Gastrointestinal Surgery, Second Affiliated Hospital of Zhejiang University, School of Medicine, Hangzhou, China

**Keywords:** transabdominal preperitoneal (TAPP), totally extraperitoneal (TEP), inguinal hernia, laparoscopic inguinal hernia repair (LIHR), surgery

## Abstract

**Background:**

Transabdominal preperitoneal (TAPP) repair and totally extraperitoneal (TEP) repair are the primary surgical methods for the treatment of adult inguinal hernia, but it remains necessary to consider which one to choose in clinical practice. Our study seeks to compare the efficacy of laparoscopic TAPP and laparoscopic TEP in the treatment of adult inguinal hernia and to explore which surgical method is a better choice.

**Methods:**

A retrospective analysis of 686 adult patients with inguinal hernia admitted to our hospital from the period January 2016 to December 2020 was conducted. According to different surgical methods, they were divided into two groups: a TAPP group (*n* = 361) and a TEP group (*n* = 325). These two groups of patients were statistically analyzed, and the operation time, postoperative pain, postoperative hospital stay length, postoperative complications, and recurrence rate were compared between them.

**Results:**

There were no significant differences in postoperative hospital stay, complications, and the recurrence rate between the two groups (*p *> 0.05). The duration of operation in the TEP group was significantly shorter than that in the TAPP group, and the difference was statistically significant (*p *< 0.001); in terms of postoperative pain, the TEP group fared better than the TAPP group, and the difference was statistically significant (*p *< 0.001).

**Conclusion:**

TAPP and TEP are safe and effective surgical methods in the treatment of adult inguinal hernia. However, compared with TAPP, TEP can significantly shorten the operative time, reduce intraoperative trauma, and limit postoperative pain in the treatment of adult inguinal hernia. Furthermore, it does not increase the rate of complications or recurrence, so it is worth popularizing.

## Introduction

Inguinal hernia is one of the common clinical types of hernia (1) and refers to an external abdominal hernia that occurs in the groin area—that is, the organs or tissues in the abdominal cavity protrude to the body surface through a congenital or acquired defect existing in the abdominal wall of the groin area. The known types of inguinal hernia include indirect hernia, direct hernia, femoral hernia, compound hernia, and so on (2–4). If an inguinal hernia is not treated in time, it may lead to serious complications.

Surgery is the main method for the treatment of inguinal hernia (5). According to statistics, there are more than 20 million cases of inguinal hernia treated by surgery in the world annually (2, 6–8). With the development of laparoscopic technology, laparoscopic inguinal hernia repair (LIHR) has gradually been adopted in clinical practice. As a micro-innovative surgical approach, LIHR provides more options for the treatment of inguinal hernia (9, 10). Compared with traditional open surgery, minimally invasive surgery can relieve patients’ surgical trauma, reduce the risk of postoperative complications, and limit patients’ postoperative pain, making it the preferred surgical method for the clinical treatment of inguinal hernia at present (11).

The preferred LIHR operations recommended by the international guidelines for adult inguinal hernia management are transabdominal preperitoneal (TAPP) inguinal hernia repair and totally extraperitoneal (TEP) inguinal hernia repair (2). At present, some controversies about the choice of surgical method persist. In order to explore the efficacy and safety of the two aforementioned surgical methods for the treatment of adult inguinal hernia, our clinical data are analyzed and summarized as follows.

## Methods

### Study Design

A total of 686 adult patients with inguinal hernia admitted to our hospital from January 2016 to December 2020 were enrolled, including 621 males and 65 females, aged 58.37 ± 13.35 years in the TAPP group and 61.58 ± 11.29 years in the TEP group. There were 353 cases of indirect hernia, 221 cases of direct hernia, 37 cases of femoral hernia, and 75 cases of composite hernia. According to different surgical methods, 686 patients were divided in such a way that 361 patients were included in the TAPP group and 325 patients in the TEP group. Gilbert types included 11 cases of type I, 179 cases of type II, 164 cases of type III, 116 cases of type IV, 120 cases of type V, 66 cases of type VI, and 30 cases of type VII (Table 1). Inclusion criteria were as follows: (1) Definite diagnosis of inguinal hernia: We usually performed B-ultrasound of the inguinal mass and CT scan for recurrent hernias or large scrotal hernia to confirm the diagnosis of hernia. (2) Laparoscopic surgery was performed with TAPP or TEP: We included both small and large hernias in this study as long as they were not within the exclusion criteria. The exclusion criteria included the following: (1) those who had a history of lower abdominal surgery; (2) large scrotal hernias; (3) incarcerated hernias; (4) coagulopathy; (5) severe cardiovascular and cerebrovascular diseases; (6) immune system diseases; (7) severe liver, kidney, and lung dysfunction; (8) and those who could not tolerate pneumoperitoneum or general anesthesia.

**Table 1:** Baseline characteristics of patients in the TAPP group vs. TEP group.

Characteristics	TAPP group (*n *= 361)	TEP group (*n *= 325)	*t*/*χ*^2^ value	*p*-value
Gender (*n*)			0.220	0.639
Male	325	296		
Female	36	29		
Age (mean ± SD, years)	58.37 ± 13.35	61.58 ± 11.29	−3.404	0.001
Hernia type (*n*)			22.172	0.001
Direct hernia	93	128		
Indirect hernia	214	139		
Femoral hernia	14	23		
Complex hernia	40	35		
Gilbert typing (*n*)			23.749	0.001
I	8	3		
II	103	76		
III	103	61		
IV	53	63		
V	49	71		
VI	35	31		
VII	10	20		

*TAPP, transabdominal preperitoneal; TEP, totally extraperitoneal.*

### Surgical Procedures

The operation was performed by a team of experienced fixed surgeons according to the operation guidelines and technical points of laparoscopic inguinal hernia surgery.

#### Transabdominal Preperitoneal

**Figure 1:**
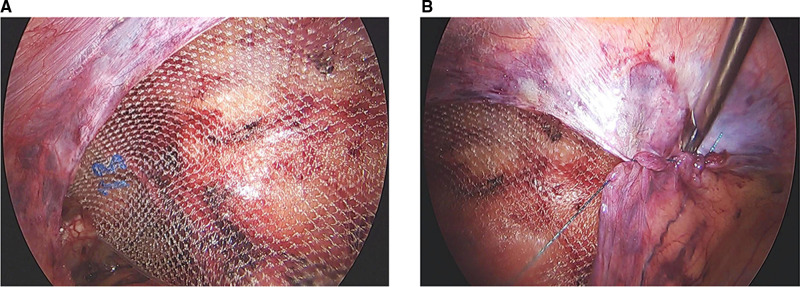
(**A**) The 3D max mesh was placed to cover the myopectineal orifice after the anterior peritoneal space was separated in transabdominal preperitoneal (TAPP). (**B**) The incised peritoneum was sutured with agnail stitches in TAPP.

After general anesthesia, the patient was placed in the supine position with the head low and foot high at 10°–15°. A 10 mm Trocar was placed 0.5–1 cm below the umbilicus as an observation hole, and the pneumoperitoneum pressure was 12–15 mmHg. The other two 5 mm Trocars were located at the level of umbilicus at the outer edge of the rectus abdominis on the affected side and below the umbilicus at the outer edge of the rectus abdominis on the healthy side, respectively (parallel to the umbilicus for bilateral hernia). Abdominal cavity and inguinal area were explored to determine the type and classification of hernia, and occult hernia was detected on the contralateral side. About 2 cm above the hernia ring, the peritoneum was cut in an arc from the medial umbilical ligament to the anterior superior iliac spine, and the medial pubic bladder space (Retzius space) and the lateral iliac fossa space (Bogros space) were dissected. The internal spermatic fascia was cut, and the hernia sac was separated and retracted (transected if necessary). We continued to dissociate the hernia sac and its continuous peritoneum at high position and dissect the spermatic cord and vas deferens at ultrahigh position. The length of the peritoneal reflection from the deep inguinal ring was 6–8 cm to achieve spermatic cord abdominal wall. The Bard 3D MAX 15 cm × 10 cm mesh was inserted to completely cover the whole myopectineal orifice (the medial side exceeded the midline by 1–2 cm, the lateral side reached inside and above the anterior superior iliac spine, and the upper edge exceeded the hernia ring defect by 3 cm. The inner lower edge was 2 cm below the pectineal ligament, and the outer lower edge was flush with the peritoneal reflected line). The peritoneum was closed by continuous suture with the barb line, and the puncture hole in the abdominal wall was sutured (Figure 1).

#### Totally Extraperitoneal

**Figure 2:**
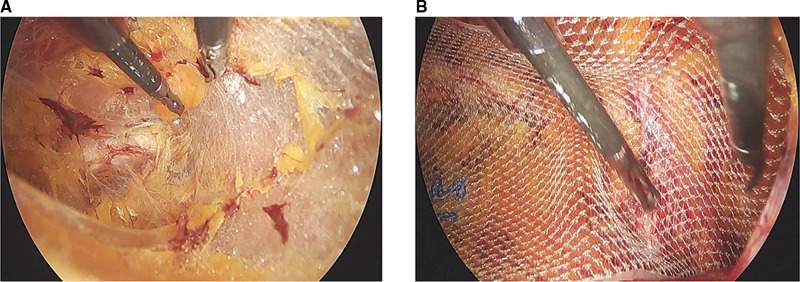
(**A**) The Retzius space was separated in totally extraperitoneal (TEP). (**B**) The 3D max mesh was placed to cover the myopectineal orifice after the anterior peritoneal space was separated in TEP.

Anesthesia and position were the same as TAPP. A transverse incision of about 1.5–2 cm was made at 0.5–1 cm below the umbilicus. The skin and subcutaneous tissue were retracted with the hook to expose the anterior sheath of rectus abdominis. The anterior sheath of rectus abdominis was cut at 0.2–0.5 cm on the affected side. The rectus abdominis muscle was retracted to both sides with a small retractor and the posterior sheath was exposed. The posterior sheath was slightly expanded and separated bluntly into the space between the dorsal side of the rectus abdominis muscle and the posterior sheath, and the space was widened by blunt separation. A 10- mm trocar was placed and filled with CO_2_ at a pressure of 12–15 mmHg. The laparoscopic lens was inserted into the pubic bladder space along the posterior sheath, pushed down and passed through the fascia transversalis, and entered the preperitoneal space. The peritoneal space was created with blunt dissection using the endoscope. In addition, a 5- mm Trocar was placed in the upper and lower 1/3 of the midline from umbilicus to pubic symphysis. After the operation space was established, the procedures from the free space to the insertion of the mesh were the same as TAPP. After the surgery, CO_2_ was slowly discharged by pressing the lower edge of the mesh under direct vision, and the puncture hole was closed by suture. If there was gas leakage to the abdominal cavity, it was released by using the pneumoperitoneum needle. If any doubts were cast during the operation, if necessary, the abdominal cavity was entered to explore whether the peritoneum was damaged, whether the mesh was flattened, and whether the hernia contents were damaged (Figure 2).

### Outcomes

(1) Perioperative indices, including operation time, postoperative hospital stay, and postoperative pain score, were compared between the two groups. (2) Complications: Postoperative incision infection, seroma, postoperative fever, postoperative hemorrhage, chronic pain, uroschesis, and other complications were compared between the two groups. (3) Recurrence rate: The patients were followed up for 1 year after surgery to compare the recurrence rate of inguinal hernia between the two groups.

### Statistical Analysis

The data were analyzed by using SPSS26.0 software (SPSS Inc., Chicago, IL, USA). Continuous variables were expressed as mean ± standard deviation, and the *t*-test was performed. Categorical variables were analyzed by using the Pearson chi-square test or Fisher’s exact test. Differences with *p *< 0.05 were considered statistically significant.

## Results

### Perioperative Indicators

The duration of surgery in the TEP group was shorter than that in the TAPP group, and the postoperative pain score was lower than that in the TAPP group, with statistically significant differences (*p *< 0.001), while there was no statistically significant difference in the length of hospital stay between the two groups (*p *> 0.05) (Table 2).

**Table 2:** Perioperative clinical data of patients in the TAPP group vs. TEP group.

Variables	TAPP group (*n* = 361)	TEP group (*n* = 325)	*t* value	*p*-value
Operation time (mean ± SD, min)	81.99 ± 39.17	60.22 ± 23.76	8.897	<0.001
Postoperative hospital stay (mean ± SD, day)	2.35 ± 1.36	2.28 ± 1.00	0.748	0.455
VAS pain score 24 h after surgery (mean ± SD)	2.45 ± 0.75	2.24 ± 0.56	4.177	<0.001

*VAS, Visual Analogue Scale/Score; TAPP, transabdominal preperitoneal; TEP, totally extraperitoneal.*

### Incidence of Complications

There was no significant difference in the incidence of incision infection, seroma, postoperative fever, postoperative hemorrhage, chronic pain, uroschesis, and other related complications between the two groups (*p *> 0.05) (Table 3).

**Table 3:** Postoperative complications of patients in the TAPP group vs. TEP group.

Complications	TAPP group (*n* = 361)	TEP group (*n* = 325)	RR	95% CI	Prevention Fraction (%)	*χ*^2^ value	*p*-value
Surgical site infections	0	0	–	–	–	–	–
Seroma	3	1	0.37	0.04–3.54	63.0	0.157	0.692
Postoperative hemorrhage	1	2	2.22	0.20–24.39	55.0	0.008	0.927
Chronic pain	2	1	0.56	0.05–6.10	44.5	0.000	1.000
Uroschesis	8	5	0.69	0.23–2.10	30.6	0.422	0.516
Epididymitis	1	0	–	–	–	–	1.000^a^
Atrial fibrillation	0	2	–	–	–	–	0.224^a^
PE/DVT	1	1	1.11	0.07–17.69	10.0	0.000	1.000
Postoperative fever	3	1	0.37	0.04–3.54	63.0	0.157	0.692
Recurrence	1	1	1.11	0.07–17.69	10.0	0.000	1.000

*PE, pulmonary embolism; DVT, deep vein thrombosis; TAPP, transabdominal preperitoneal; TEP, totally extraperitoneal; CI, confidence interval; RR, recurrence rate*.

*^a^* *Fisher’s exact test.*

### Recurrence Rate within 1 Year after Surgery

In the TAPP group, 1 of 361 patients had recurrence within 1 year after surgery, and the recurrence rate was 0.28%. Among 325 patients in the TEP group, 1 patient had recurrence within 1 year after surgery, and the recurrence rate was 0.31%. There was no statistically significant difference between the two groups (*χ*^2^ = 0.000, *p *= 1.000) (Table 3).

## Discussion

With the continuous development of laparoscopic surgery, the proportion of LIHR procedures has gradually increased, and LIHR has become an important surgical treatment for inguinal hernia (12, 13). Compared with previous open tension-free repair operations, it has the advantages of a smaller incision, less pain, and a quicker return to normal activities (14–19). Among the surgical methods used in clinical practice, the most commonly used ones are laparoscopic TAPP and laparoscopic TEP (20, 21). However, there is still controversy about which of these two approaches comes first in academia. In this study, the statistical analysis of TAPP and TEP surgical methods showed that there were no significant differences in the postoperative hospital stay length, complications, and recurrence rate between the two groups (*p *> 0.05). Compared with the TAPP group, the TEP group had a significantly shorter operation time, and the difference was statistically significant (*p *< 0.001). This result may be due to the fact that TAPP requires a peritoneal incision and final suture during the operation, which increases the number of procedural steps and prolongs the operation time. In terms of postoperative pain, the TEP group fared better than the TAPP group, and the difference was statistically significant (*p *< 0.001). This outcome may be due to the relatively short duration of TEP surgery, lack of accessing the abdominal cavity, and no peritoneal sutures.

The placement position of the mesh during TAPP and TEP is the same, with both involving complete coverage of the whole range of the myopectineal orifice. However, these procedures have their own unique characteristics as well. TAPP surgery requires entering the abdominal cavity to open and close the peritoneum. The surgical operation space is large, and the anatomical structure is easy to identify. The operation technology is relatively simple, but it is easy to affect the abdominal organs during the procedure. For example, the imprecise peritoneal suture is likely to lead to abdominal adhesion. In contrast, TEP surgery allows the preperitoneal space to be separated completely through the extraperitoneal cavity without entering the abdominal cavity (22–24); then, the mesh is inserted into this space. Due to the operation being carried out outside the peritoneum, it has little effect on the viscera in the abdominal cavity. Technically, TEP is more reasonable, given that it is completed without entering the abdomen, but its operation space is small, and the identification of anatomical structures is relatively complex (25). If the operation is not completed properly, the peritoneum may easily be damaged. Furthermore, the surgical space is narrowed after the gas enters the abdominal cavity, increasing the difficulty of the operation and prolonging the learning curve (26).

As for how to make an appropriate choice between the two surgical methods, after analyzing the clinical data of our center, we suggest that the choice of the surgical method generally depends upon the experience of the surgeon in combination with the guidelines to be followed. We contend that TAPP is relatively simple and suitable for beginners and all types of hernia, but it should be used with caution for treating patients with obvious inferior abdominal adhesion on the affected side. The TEP surgery is difficult and should be performed by surgeons skilled in LIHR. TEP is preferred for bilateral hernia (27). In the treatment of patients with irreducible hernia, recurrent hernia, a long course of disease, or a large hernia sac, TAPP can facilitate the reduction of hernia contents and reveal whether there is intestinal necrosis and other intra-abdominal conditions. Its safety is also better than that of TEP (28). In addition, if the performance of TEP surgery is found to be difficult or if it fails, it can be transformed to TAPP.

In general, TEP has certain advantages over TAPP; however, it is difficult for beginners to perform this operation. Successful establishment of the extraperitoneal space is crucial for ensuring TEP success. Particularly when entering the Bogros space from the Retzius space, hernia sac stripping is prone to causing peritoneal damage. After the peritoneum is damaged, gas enters the abdominal cavity and elevates the peritoneum, resulting in a more narrow surgical operation space and affecting the surgical field of vision. This is a common reason for the failure of TEP surgery in the initial stage. Some studies have reported that the incidence of postoperative complications associated with TEP may be higher than that affiliated with TAPP. This may be related to the inexperience and limited operation skills of surgeons performing the TEP surgery. The results of our study showed that there was no statistically significant difference in postoperative complications between the TAPP group and the TEP group (*p *> 0.05). One case of recurrence occurred in each group during the first year of follow-up, and the difference between groups was not statistically significant (*p *> 0.05). In both cases, the recurrence site was the same as the original surgical site. This low number of patients who experienced recurrence may be related to the fact that the surgeons in this study were all from an experienced team. Based on our clinical experience, we suggest that TEP surgery should be carried out gradually after gaining some amount of experience on the basis of TAPP surgery. Through standard and corresponding surgical skills training, surgical safety can be significantly improved and related complications can be reduced (29).

There are still several limitations that need to be considered in the current study. First, this was a retrospective study with a follow-up period of only 1 year. There are also some differences with patient age and hernia typing in the baseline characteristics. Long-term follow-up analysis of patients should be continued, and more data are required to reduce or eliminate such differences. Besides, a larger-scale prospective randomized controlled trial with the goal of providing higher-level evidence in the future should be carried out.

## Conclusion

In conclusion, TAPP and TEP are both safe and feasible in the treatment of inguinal hernia, but TEP has more advantages than TAPP, so we prefer to recommend TEP. In clinical work, we should make a reasonable choice according to the specific situation of patients and the clinical experience of surgeons.

## Data Availability

The original contributions presented in the study are included in the article/Supplementary Material; further inquiries can be directed to the corresponding author/s.
